# 

**Published:** 1895-06-15

**Authors:** 


**Notices of Births, Deaths, and Marriages** are inserted in this column at a charge of 2s. 6d. for an announcement not exceeding four lines.

**Notice to Advertisers and Subscribers.**
All Advertisements, Orders for Copies of The Hospital and Business Communications should be addressed to **The Manager, NOT TO THE EDITOR,** The Hospital, 428, Strand, London, W.C.
The Cost of Subscription, post free, is as follows:—Per week, 2½d.; per quarter, 2s. 8d.; per half-year, 5s. 3d.; per annum, 10s. 6d. Foreign:—Per Annum, 15s. 2d.; payable, in all cases, in advance.

### NOTICE TO CORRESPONDENTS.

All MS., Letters, Books for Review, and other matters intended for the Editor should be addressed The Editor, The Lodge, Porchester Square, London, W.
The Editor cannot undertake to return rejected MS., even when accompanied by stamped directed envelope.

**"Burdett's Hospital Annual," 1895,**
Now Ready.
Sixth year of publication. 950 pp. Scarlet cloth, gilt lettered. Price 5s. A most complete guide to British, Colonial, Indian, and American Hospitals, Dispensaries, Nursing and Convalescent Institutions, and Asylums. A few copies of the "Annual" for 1894 may still be obtained on application to the Publishers, The Scientific Press (Limited), 428, Strand, London, W.C.

**NOTICE.—Vol. XVII. of "The Hospital" (October, 1894, to April, 1895), in handsome cloth binding, is now on sale at the Office of this Journal. Price 6s. Cases for binding the Numbers contained in this Volume, 1s. 6d. Index to Vol. XVII., 2½d., post free.**
**The Monthly Part for June is now ready, price 6d.; by post, 8d.**

### Scraps and Gleanings.

Hong Kong papers tell us that the plague has broken out in the city.

Lord Blythwood has laid the foundation-stone of the Glasgow Samaritan Hospital for Women.

A special service for the East London Nursing Society was held in St. Paul's Cathedral on Tuesday in last week.

At Villiask, in Eastern Siberia, a leper colony has been established, where about forty afflicted persons are at present housed.

Two legacies of £100 have been received by the Edinburgh Royal Infirmary from James Russell and Miss Marian Campbell.

The sum of £54 was handed over to the Abergavenny Cottage Hospital as the outcome of Mr. Studt's entertainment in its honour.

The Duchess of Teck paid a visit to the Chelsea Hospital for Women on Tuesday, 28th inst., and opened a ward named in her honour the "Mary Adelaide Ward."

The ceremony of opening the new Caxton wing of the Morley House Convalescent Home, St. Margaret's, Dover, has just been performed by the Hon. W. F. Smith, M.P.

An annual sale of fancy work, done during the year by the inmates of Mauldeth Hospital for Incurables, Manchester, has just taken place. The Lady Mayoress of Manchester opened the sale in the large central hall of the institution.

The position of affairs in connection with the Cottage Hospital at Ashford forms a burning question of the hour in local matters, and a great deal of anxiety exists as to whether the course of events will lead to the closing of the institution.

A Bill has been introduced in America to provide that candidates studying medicine must have completed a full academic course, instead of the hitherto prescribed three years' course. This will obviate the necessity of taking Regent's examinations.

At the last meeting of the Shildon Urban District Council the question of the provision of hospitals for infectious diseases was again under discussion, and it was decided to ask the County Council to contribute to the expenses of any hospital which might be built.

We are glad to learn that the East London Church Fund, which has been carrying on its unobtrusive Evangelistic work, has received substantial financial support lately. The receipts for the past year, for example, show an advance of £400 on those of 1893.

Rheumatism in Malta continues to be treated, in severe cases, by bee stings, a method which has preserved its reputation for a long time. The antidote is said to give great relief to sufferers, though on first hearing it does not recommend itself so well to the uninitiated.

The building fund of the New Samaritan Hospital at Glasgow is still about £3,000 short of the sum required to meet the estimated cost of the buildings, and a further sum of £1,500 will be needed to furnish the new hospital. The directors are appealing for additional subscriptions.

A contemporary points out a new Act recently passed in America for the incorporation of pathological and anatomical associations for the advancement of medical and surgical science and the legalisation of dissection of paupers. The Governor of New Jersey has duly signed this Bill.

The thirteenth annual demonstration and church parade of the friendly societies of the metropolis, in aid of the funds of the North-West London and University College Hospitals, took place early this month, collections on behalf of the hospitals being made all along the line of route.

The proprietors of the Liquor Carnis Company (raw beef juice preparers) wish to call attention to the fact that they have decided to substitute the initial letters "L. C. C." for Shepperson. Henceforth the correct title is "L. C. C.," until now known as "Shepperson's Meat Juice."

The medical superintendent of the Ladywell Sanatorium, Salford, in issuing his annual record of the work done at the hospital during the year, states that the total number of cases admitted to the sanatorium and the Mode Wheel Hospital during the year ending December, 1894, was 1,103.

A somewhat gruesome fact is divulged by the *Boston Medical and Surgical Journal*, which states that the Commission into the prevalence of contagious diseases in the New York schools brought forward the fact that, out of 112 school trustees, 20 per cent. are undertakers! No inference need necessarily be drawn, but, as a statistic, it is curious.

Dr. Franck has brought under the notice of the Polytechnic Society of Berlin a new means of disinfecting wells. He has employed the same himself with signal success. His method is to suspend an earthenware dish containing 50 to 100 grammes of bromide, which forms a dense vapour, filling the well, and is in this way absorbed by the water.

An American religious journal calls our attention to some investigations made by medical men into the so-called "gold" cure for drunkenness. Out of 534 cases thus investigated, of which fullest particulars have been had, 275 are reported to be entirely cured, 251 relapsing. If these statistics are to be relied upon, this gives us a cure of 50 per cent.

The annual sale of work for the benefit of the patients of the Broomhill Home, Glasgow, took place the end of last month. The Duchess of Montrose was present on this occasion, and also opened a new wing which the directors have been enabled to add to the building from the proceeds of the great bazaar held in 1893 in St. Andrew's Hall, Glasgow.

In the fourteenth yearly report of St. Michael's Orphan Home, at Frampton Cotterell, we are glad to see that the financial difficulties, which were the main features of last year's report, have materially decreased, owing to the great efforts made to reduce the debt. This home is one fulfilling a much-needed want, as it is open for the reception of orphan, destitute, friendless, illegitimate, and delicate children.

Steps are being taken by the Oxford Radcliffe Infirmary to augment the funds of that institution. It is proposed to start a personal canvass in the city, and a circular has been issued pointing out the pressing needs of a further subscription list. The income the infirmary derives from its capital fund is considerably less than one-third of the necessary expenditure, the remainder of which, therefore, has to be met by annual subscriptions.

### Books Received.

Digby, Long, and Co.

"The Hon. Mrs. Spoor." Arabella Kenealy.

John Wright and Co. (Bristol).

"The Eye in its Relation to Health." Chalmers Prentice, M.D.

Charles Griffin and Co.

"Essays in Heart and Lung Disease." Arthur Foxwell, M.D.

"Official Year Book of the Scientific and Learned Societies of Great Britain and Ireland."

**Periodicals and Pamphlets.**—The Sun, Popular Science News, Electrical Review, Christian World, London, Homœopathic Review, Nature, Westminster Review, Public Health, Invention. Indian Medico-Chirurgical Review, The Planter, The Corpuscle, The English Illustrated Magazine, Sunday at Home, Leisure Hour, Girl's Own Paper, Boy's Own Paper.

### The Student of Medicine.

### APPOINTMENTS

W. H. Blake, L.R.C.P.Lond., M.R.C.S.Eng., appointed Medical Officer for the West Wickham District of the Beckenham Union. F. H. Cooke, M.R.C.S., L.R.C.P.Lond., appointed Medical Officer for the Sixth District of the Lexden and Winstree Union. H. B. Gladstone, M.B., C.M.Edin., appointed House-Physician to the Royal Hospital for Diseases of the Chest, City Road, E.C. L. Houghton, M.R.C.S.Eng., L.R.C.P.Lond., appointed Medical Officer for the Looe District of the Liskeard Union. T. B. Hyslop, M.D.Edin., appointed Lecturer on Mental Diseases in St. Mary's Hospital Medical School. J. Miller, M.B., C.M., D.P.H., appointed Medical Officer of Health for the Burgh of Largs. E. Riding, M.R.C.S.Eng. and L.S.A., appointed Medical Officer of Health to the Repton Rural District Council. W. Robinson, M.D., M.S.Durh., F.R.C.S.Eng., appointed Honorary Surgeon to the Sunderland and North Durham Eye Infirmary. H. W. Scratchley, L.R.C.P.Lond., M.R.C.S.Eng., appointed Medical Officer for the Fifth Districts of the Poole Union. R B., Smith, L.R C.P.Edin., L.R.C.S.I., appointed Medical Officer for the Wigston Parva District of the Hinckley Union. Dr. Thomas, appointed Medical Officer for the Polperro District of the Liskeard Union. J. H. Thompson, L.R.C.P.I., L.R.C.S.Edin., appointed Medical Officer for the Mytholmroyd District of the Todmorden Union. M. C. Weeks, appointed Medical Officer for the Pinchbeck District of the Spalding Union. A. P. Wells, B.A.Cantab., L.R.C.P.Edin., appointed Medical Officer and Public Vaccinator for the Beckenham District of the Beckenham Union.

### THE STUDENT OF MEDICINE—*continued.*

### VACANCIES.

The following vacancies are announced this week, the amount of salary in each case being given after the name of the institution:

**Resident Medical Officers.**

Eve ina Hospital for Sick Children, Southwark Bridge Road, S.E.—Senior and Junior Resident Medical Officers. Salaries £70 and £50 per annum respectively. Applications to the Committee of Management by June 15th.

Flintshire Dispensary.—Resident House Surgeon. Salary £120 a year, with furnished house, rent, and taxes free, also coal, light, water, and cleaning, or in lieu thereof the sum of £20 per annum. A knowledge of Welsh is desirable. Application to Thos. Thomas, Secretary, Board Room, Bagillt Street, Holywell, North Wales, by July 17th.

Hospital for Sick Children, Great Ormond Street, Bloomsbury. W.C.—House Surgeon to the Out-patients. Appointment for six months, but eligible for re-election for a further six months. Salary 25 guineas. Applications to the Secretary by June 18th.

Kent County Lunatic Asylum, Barming Heath near Maidstone.—Fourth Assistant Medical Officer and Pathologist; unmarried. Salary £175 per annum, rising £5 a-year, with furnished quarters, attendance, coal, gas, garden produce, and washing. Appointment for two years. Applications to F. Pritchard Davis, Superintendent, by June 18th.

Manchester Royal Infirmary.—Resident Medical Officer; not under 25 years of age, unmarried, and doubly qualified. Appointment for one year, but eligible for re-election. Salary £150 per annum, with board and residence. Applications to the Chairman of the Board by June 22nd.

North-Eastern Hospital for Children.—Junior House Physician; doubly qualified. Appointment for six months. No salary, but board and lodging (including washing) provided. Applications to the Secretary at 27, Clement's Lane, E.C., by June 21st.

Parish of Lambeth.—Medical Officer for the Workhouses and Infirmary; doubly qualified; must devote his whole time to the duties of the office. Salary £300 per annum, with furnished house, and allowance of coals, gas, and water. Applications to W. B. Wilmot, Clerk, Guardians' Board Room and Offices. Brook Street, Kennington, S.E., by June 17th.

Radcliffe Infirmary, Oxford.—House Physician. Appointment for six months. Salary at the rate of £60 per annum, with board and lodging. Applications, on printed forms provided, to be sent to the Secretary before June 19th.

St. Pancras and Northern Dispensary, 126, Euston Road. N.W.—Resident Medical Officer; doubly qualified. Salary £105 per annum, with residence and attendance. Applications to the Honorary Secretary, H. P. Bodkin, 23, Gordon Street, Gordon Square, W.C., by June 18th.

Royal Berks Hospital, Reading.—Assistant Medical Officer. Board and lodging provided; no salary. Appointment for six months. Applications to the Secretary before June 15th.

West London Hospital, Hammersmith Road, W.—House Physician and House Surgeon. Appointments for six months. Board and lodgings provided. Applications to R. J. Gilbert, Secretary-Superintendent, by June 19th.

**Non-Resident Medical Officers.**

Kilburn, Maida Vale, and St. John's Wood Dispensary.—Vacancy on the Honorary Medical Board. Applications to the Secretary, 13, Kilburn Park Road, N.W., by June 19th.

London County Council.—Pathologist to the London County Asylums. Salary £700 per annum, and travelling expenses. Applications (on forms provided), endorsed "Applications for Pathologist," to R. W. Partridge, Clerk of the Asylums Committee, 21, Whitehall Place, S.W., by June 17th.

London Hospital, Whitechapel, E.—Medical Electrician. Applications to the House Governor by June 29th.

Trinity College, Dublin.—Lecturer in Pathology. Applications to the Registrar of the University by June 15th.

### UNIVERSITIES AND COLLEGES.

UNIVERSITY OF LONDON.

M.B. Pass Examination, May, 1895.—Pass List.

First Division.—J. O. Harvey, St. Bartholomew's Hospital; J. R. Hickinbotham, Mason College, and General Hospital, Birmingham; W. H. Pollard, St. Bartholomew's Hospital; H. M. Rigby, London Hospital; A. Tait, Yorkshire College, and General Infirmary, Leeds; W. Turner, King's College; and J. E. Waite, University College.

Second Division.—G. J. Branson, B.A., Mason College; F. M. Burnett, St. Bartholomew's Hospital; A. W. R. Cochrane, St. Bartholomew's Hospital; W. H. W. Elliott, Guy's Hospital; P. C. Fenwick, St. Thomas's Hospital; G. W. Gostling, University College; W. E. L. Horner, University College; S. P. James, St. Mary's Hospital; R. L. Jones, University College; Harriet Edith Florence Knight, London School of Medicine, and Royal Free Hospital; J. Mace, Yorkshire College, and General Infirmary, Leeds; H. F. Mantell, St. Mary's Hospital; A. A. Martin, St. Mary's Hospital; L. J. Miskin, St. Thomas's Hospital; R. H. Norman, Westminster Hospital; C. Oldfield, Yorkshire College; B. A. Richmond, B.Sc., Guy's Hospital; C. D. D. Roberts, Mason College, and General Hospital, Birmingham; G. N. O. Slater, St. Bartholomew's Hospital; E. Sly, King's College; T. A. Starkey, University College; J. R. Steinhaeuser, Guy's Hospital; and Helen Swatman, London School of Medicine and Royal Free Hospital.

### The Hospital.

AN

### Institutional Journal of the Medical Sciences and Hospital Administration.

**Weekly 2d.** **Monthly 6d.**

WITH AN EXTRA NURSING SUPPLEMENT OF 20 PAGES.

Published Weekly. Price **2d.** Publishing and Editorial Offices: 428, Strand, London, W.C.

The success which has attended the publication of "THE HOSPITAL" newspaper, and its unique circulation amongst the staff of the hospitals and medical institutions, not only in this country, but in Greater Britain, India, and the United States of America, have encouraged the Editors to enlarge the paper, with the view of making it more generally useful to the great body of medical practitioners.

Dr. Pye Smith, in the Harveian Oration this year, pointed out that "there is all the difference between learning the present conclusions as they stand in the last edition of a text-book or compendium, and tracing the steps by which our present knowledge has been reached." The conductors of "THE HOSPITAL," in the new departure which they are making in medical journalism, have determined to reduce this exhortation to practice by securing the co-operation of eminent members of the medical profession who are also able writers and close observers, who will describe the progress of the science of medicine, and trace the steps by which our present knowledge has been reached.

With this view arrangements have been made for a series of articles on

"MODERN THERAPEUTICS," by Sir Benjamin Ward Richardson, M.D., F.R.S.;

On "MODERN PROGRESS IN OPHTHALMIC MEDICINE AND SURGERY," by Mr. R. Brudenell Carter, F.R.C.S., Consulting Ophthalmic Surgeon to St. George's Hospital;

On "SOME ASPECTS OF MODERN PSYCHOLOGY," by Dr. Clouston, F.R.C.P., Edin., Lecturer on Mental Diseases to the University of Edinburgh; and

On "MODERN BIOLOGY," by Mr. J. Bretland Farmer, M.A., F.L.S., Assistant Professor of Biology Royal College of Science South Kensington; in addition to articles on other subjects, which will be treated by physicians, surgeons, and scientists of eminence.

And for many other articles of special interest, all written by experts.

One of the new departments in "THE HOSPITAL" is entitled "MEDICAL PROGRESS," and provides the profession with a concise, authoritative, and readable epitome of the most recent advances in medical science.

The scheme of the journal is entirely original, and it in no way resembles the ordinary medical journals.

In addition to original contributions upon treatment, practice, and therapeutics, each number contains a comprehensive record of progress in one or more branches of medicine, surgery, laryngology, obstetrics, gynæcology, ophthalmology, and other special departments, so as to secure the whole field of medicine being covered, in the widest and fullest meaning of the term.

These articles are not prepared from a critical or partizan point of view, but are made to embrace the chief points of every paper and contribution of importance, so that each worker, in his own particular branch will here find a complete record of all the work done by others, wherever trained practitioners are trying to solve the problem of disease.

It may further be added that the department of "MEDICAL PROGRESS" will be strengthened by ample and precise references to the original papers and sources of information.

No expense has been, or will be, spared to make "MEDICAL PROGRESS AND HOSPITAL CLINICS" exhaustive and complete.

The practical value of this new departure in medical journalism must be patent to every practitioner, and it will fill a place unoccupied by any other publication hitherto produced in the English language.

The true interests of the majority of medical practitioners are much concerned with the wise ADMINISTRATION OF HOSPITALS and medical institutions. "THE HOSPITAL" deals with the various questions involved in all their bearings, and so helps to form and lead medical and public opinion on these subjects.

The WOKSHOP SECTION contains plans of all the most recent improvements and developments in hospital construction, including drainage and sanitation, matters which seriously affect the welfare of the patients, and are becoming every day of more importance and interest to the medical profession. The reports on hospital administration by "THE HOSPITAL'S" special commissioner, containing an account of the present condition and work of the chief British and foreign hospitals, have already excited a wide interest, and have proved not only popular but instructive to all who are interested in the subject.

"THE HOSPITAL" has already acquired an influence and circulation which has given it a foremost place in medical journalism, and this fact, coupled with the representations which have been made to the Publishers by influential and representative members of the medical profession, point to the conclusion that the many new developments now determined upon, and especially the department devoted to "MEDICAL PROGRESS AND HOSPITAL CLINICS," will command a warm and extended welcome, and so secure that "THE HOSPITAL," which already commands the largest *bona fide* sale of any English medical journal, will speedily possess the greatest circulation also.

"THE HOSPITAL": EDITORIAL AND PUBLISHING OFFICES, 428, STRAND, LONDON, W.C.

### THE HOSPITAL.

AN INSTITUTIONAL JOURNAL OF THE MEDICAL SCIENCES AND HOSPITAL ADMINISTRATION.

WEEKLY, 2d. **SUBSCRIPTION FORM.** MONTHLY, 6d.

TO THE PROPRIETORS OF "THE HOSPITAL,"
428, STRAND, LONDON, W.C.

*Kindly enter my name as a Subscriber to* **"The Hospital"** *for* {TWELVE MONTHS / SIX MONTHS / THREE MONTHS}

*the price for the same being enclosed, viz.,* {TEN SHILLINGS AND SIXPENCE * / FIVE SHILLINGS AND THREEPENCE / TWO SHILLINGS AND EIGHTPENCE} *including postage.*

WRITE CLEARLY. *Name* ____________________

*Address* ____________________

____________________

*Date* ____________________

* Foreign rates on application.

